# An Untargeted Lipidomic Analysis Reveals Depletion of Several Phospholipid Classes in Patients with Familial Hypercholesterolemia on Treatment with Evolocumab

**DOI:** 10.3390/biomedicines9121941

**Published:** 2021-12-17

**Authors:** Andrea Anesi, Alessandro Di Minno, Ilenia Calcaterra, Viviana Cavalca, Maria Tripaldella, Benedetta Porro, Matteo Nicola Dario Di Minno

**Affiliations:** 1 Fondazione Edmund Mach Research and Innovation Centre, Food Quality and Nutrition Department, Via E. Mach, 1, 38010 S. Michele all’Adige, Italy; andrea.anesi@fmach.it; 2 Dipartimento di Farmacia, Università degli Studi di Napoli “Federico II”, 80131 Napoli, Italy; 3 CEINGE-Biotecnologie Avanzate, Università degli Studi di Napoli, 80131 Napoli, Italy; 4 Dipartimento di Medicina Clinica e Chirurgia, Università degli Studi di Napoli “Federico II”, 80131 Napoli, Italy; ileniacalcaterra@hotmail.it (I.C.); mariatripaldella@gmail.com (M.T.); 5 Centro Cardiologico Monzino, IRCCS, 38010 Milano, Italy; viviana.cavalca@ccfm.it (V.C.); benedetta.porro@ccfm.it (B.P.); 6 Dipartimento di Scienze Mediche Traslazionali, Università degli Studi di Napoli “Federico II”, 80131 Napoli, Italy; dario.diminno@hotmail.it

**Keywords:** PCSK-9, untargeted lipidomics, familial hypercholesterolemia

## Abstract

Rationale: Familial hypercholesterolemia (FH) is caused by mutations in genes involved in low-density lipoprotein cholesterol (LDL-C) metabolism, including those for pro-protein convertase subtilisin/kexin type 9 (PCSK-9). The effect of PCSK-9 inhibition on the plasma lipidome has been poorly explored. Objective: Using an ultra-high-performance liquid chromatography-electrospray ionization-quadrupole-time of flight-mass spectrometry method, the plasma lipidome of FH subjects before and at different time intervals during treatment with the PCSK-9 inhibitor Evolocumab was explored. Methods and Results: In 25 FH subjects, heterozygotes or compound heterozygotes for different LDL receptor mutations, untargeted lipidomic revealed significant reductions in 26 lipid classes belonging to phosphatidylcholine (PC), sphingomyelin (SM), ceramide (CER), cholesteryl ester (CE), triacylglycerol (TG) and phosphatidylinositol (PI). Lipid changes were graded between baseline and 4- and 12-week treatment. At 12-week treatment, five polyunsaturated diacyl PC, accounting for 38.6 to 49.2% of total PC at baseline; two ether/vinyl ether forms; seven SM; five CER and glucosyl/galactosyl-ceramide (HEX-CER) were reduced, as was the unsaturation index of HEX-CER and lactosyl—CER (LAC-CER). Although non quantitative modifications were observed in phosphatidylethanolamine (PE) during treatment with Evolocumab, shorter and more saturated fatty acyl chains were documented. Conclusions: Depletion of several phospholipid classes occurs in plasma of FH patients during treatment with the PCSK-9 inhibitor Evolocumab. The mechanism underlying these changes likely involves the de novo synthesis of SM and CER through the activation of the key enzyme sphingomyelin synthase by oxidized LDL and argues for a multifaceted system leading to vascular improvement in users of PCSK-9 inhibitors.

## 1. Introduction

Several studies emphasize the role of high levels of low-density lipoprotein cholesterol (LDL-C) as the main causative factor in the development of atherosclerosis [1,2]. Among patients with familial hypercholesterolemia (FH), those with very high levels of LDL-C exhibit enhanced prevalence of subclinical atherosclerosis and of atherosclerosis progression, both leading to a significantly higher cardiovascular (CV) risk [1,3,4]. Although statin treatment is the gold standard therapy for lipid lowering, target LDL-C levels are seldom achieved among patients with very high LDL-C [5]. Proprotein convertase subtilisin/kexin type 9 (PCSK-9) inhibitors have shown efficacy in LDL-C reduction, in the prevention of CV events, and in atherosclerotic burden regression [6,7,8].

However, while the effect of PCSK-9 inhibition on LDL-C and cholesterol metabolism has been intensively investigated, its role in the whole lipid metabolism has been little explored, despite evidence arguing for changes in lipid metabolism following administration of PCSK-9 inhibitors as being, at least in part, unrelated to cholesterol metabolism [9]. Indeed, decrease in some lipid species in human serum was not proportional to LDL-C reduction [9,10]. Furthermore, in recently published studies we performed on the same blood samples, an untargeted metabolomic analysis for gathering a global view of metabolic pathways and characterizing metabolites modified following treatment with PCSK-9i in FH subjects [11]. This raises the issue whether PCSK-9 deficiency/inhibition may impact atherosclerosis by decreasing LDL-C concentration as well as by changing the levels of other lipid classes. In FH patients with very high levels of LDL-C, we have assessed the lipidome by an untargeted lipidomic approach, before and at different time points, following treatment with the PCSK-9 inhibitor Evolocumab. 

## 2. Materials and Methods

### 2.1. Study Population and Protocol

From December 2017 to December 2018 and within the framework of LIPIGEN, a national project on familial dyslipidemia, 133 consecutive patients with very high levels of LDL-C (above the 95th percentile when compared with a sex- and age-matched general population) and normal triglyceride levels and presumed autosomal dominant transmission of FH in the family were screened in the lipid clinic of the Department of Clinical Medicine and Surgery, Federico II University Hospital, for inclusion in the present study [12]. The major inclusion criterion was eligibility for treatment with a PCSK-9 inhibitor according to the 2016 ESC guidelines [1]. Exclusion criteria were age < 18 years or >80 years; inability to understand or sign the informed consent; high level of transaminases (>3 × upper normal limit); hypertriglyceridemia (>150 mg/dL); end-stage renal disease (filtration rate <30 mL/min/m^2^); current malignant disease or malignancy in the 2 years prior to the first visit; previous exposure to PCSK-9 inhibitors; hypercholesterolemia secondary to other causes (e.g., hypothyroidism, hormone therapies, corticosteroids). During the study, the PCSK-9 inhibitor (Evolocumab 140 mg subcutaneous injections every 14 days) was given on top of the ongoing lipid-lowering treatment. After approval from the local Ethics Committee, informed consent was obtained from each patient. 

### 2.2. Blood Laboratory Parameters

Total cholesterol (TC), triglycerides (TGL), HDL cholesterol (HDL-C), LDL-C, Lipoprotein(a) (Lp(a)), small-dense LDL (sd-LDL), creatinine, AST, ALT, CPK and blood glucose were evaluated at baseline (before starting PCSK-9 inhibitor, T0) and after 4 weeks (T4w) and 12 weeks (T12w) of treatment with Evolocumab 1 mL vials with subcutaneous administration twice a week. Lp(a) was measured by an ELISA solid phase two-site enzyme immunoassay, using polyclonal antibodies raised against purified Lp(a) (Mercodia Diagnostics, Uppsala, Sweden) [13].

LDL particles separation was performed on serum samples by electrophoretic Lipoprint System (Quantimetrix Inc., Redondo Beach, CA, USA). The proportion of sd-LDL particles (subfractions 3–7) to the whole LDL area (subfractions 1–7) was calculated and expressed as the LDL score, with higher values reflecting higher sd-LDL particles content [14]. Mean LDL particle diameter was calculated based on the areas under the curve of the seven LDL species with different electrophoretic mobility [15].

### 2.3. Lipid Extraction

Frozen plasma samples were thawed in ice, and 100 µL aliquots were transferred into 10 mL borosilicate tubes. Lipids were extracted by using a slightly modified Bligh and Dyer system [16]. Briefly, 3 mL of chloroform:methanol 2:1 (*v*:*v*) containing 20 mg/L butylhydroxytoluene (BHT) was added; samples were vortexed for 10 s and sonicated in ice for 15 min in an ultrasonic bath (Falc, LabService, Milan, Italy). One milliliter of deionized water was added; samples were vortexed for 10 s and centrifuged at 3500 rpm for 10 min at 4 °C to promote phase separation. The organic phase was collected, and the aqueous phase was re-extracted by adding 2 mL of chloroform 20 mg/L BHT. The pooled organic phase was dried in a Speedvac and re-suspended in 300 µL methanol–chloroform 9:1 (*v*:*v*). Quality control samples were obtained by pooling 50 µL volumes of each sample.

### 2.4. UHPLC-ESI-Q-TOF–MS Analysis

Lipids were injected on an Agilent 1290 Infinity UHPLC (Agilent Technologies, Santa Clara, CA, USA) connected with an Agilent 6550 iFunnel Q-TOF mass spectrometer equipped with a Dual-Jet ESI source (Agilent, Milan, Italy). Lipids were separated under reverse-phase conditions on a ZORBAX Eclipse Plus C18 Rapid Resolution HD column (2.1 mm × 150 mm, 1.8 µm; purchased by Agilent, Milan, Italy) and eluted at 0.3 mL/min. Mobile phase A was acetonitrile–water 50:50 (10 mM ammonium formate, 0.1% formic acid), and B was acetonitrile–water–isopropanol 10:2:88 (10 mM ammonium formate, 0.1% formic acid). The linear gradient, starting at 65%A:35%B, reached 95%B in 20 min and 100%B in 5 min; final conditions were kept for 2.5 min to ensure complete elution of non-polar lipids. Initial conditions were reached in 0.5 min and maintained for 10 min to ensure a complete column re-equilibration.

Full-scan analysis over *m/z* 100–1200 in positive ion mode and over *m/z* 50–1200 in negative was carried out at a scan rate of 1.50 spectra/s. To avoid detector saturation, 0.2 µL of lipid extract was injected in positive ion mode and 2 µL in negative ion mode. Additional mass spectrometry settings were the following: gas temp, 230 °C; gas flow, 12 L/min; nebulizer, 35 psig; sheath gas temp, 350 °C; sheath gas flow, 12 mL/min; V cap, 3500 V for positive, 4000 V for negative; Nozzle voltage, 1000 V; fragmentor, 150 V; skimmer 1, 65 V; octopole RF peak, 750 V. MS/MS studies were carried out under the same experimental conditions using a collision energy of 30 V.

Samples were randomized prior to injection. Quality controls (QCs) were injected six times prior to analysis, every 8 samples, and at the end of the acquisition to address the instrumentation stability. Data were acquired with MassHunter software (version B.07.00; Agilent, Milan, Italy).

### 2.5. Lipid Annotation and Data Analysis

Lipids were annotated according to their *m/z* acquired in high-resolution mode; database search (Lipid Maps (www.lipidmaps.org (accessed on 10 October 2021)), Human Metabolome Database (www.hmdb.ca (accessed on 10 October 2021)), CEU Mass Mediator (www.ceumass.eps.uspceu.es (accessed on 10 October 2021)), and Mass Bank (www.massbank.jp, accessed on 10 October 2021)); and after MS/MS experiments in positive and negative ions. Lipids were denoted by head group, total fatty acyl carbon atoms and unsaturation content (e.g., PC 34:1). For ether/vinyl ether species, both species are reported when it was not possible to annotate the correct form (e.g., PC (O-36:3)/(P-36:2)).

Raw data were processed by MZmine 2.3 (www.mzmine.github.io (accessed on 10 October 2021)). In positive ion mode, phosphatidylcholine (PC) and lyso-PC (LPC), sphingomyelin (SM), diacylglycerol (DAG), triacylglycerol (TG) and cholesteryl ester (CE) were detected and annotated. In negative ion mode phosphatidylethanolamine (PE) and lyso-PE (LPE), phosphatidylinositol (PI) and lyso-PI (LPI), phosphatidylserine (PS), ceramide (CER), glucosyl/galactosyl-ceramide (HEX-CER), lactosyl-ceramide (LAC-CER) and 3-O-sulfogalactosylceramide (S-HEX-CER) were detected and annotated. Residual missing values in the data matrix were imputed using Random Forest package in R (www.r-project.org (accessed on 10 October 2021)). Data were normalized by Loess-global regression using the Normalyzer tool [17]. Multivariate statistics was carried out by MetaboAnalyst 4.0 software (www.metaboanalyst.ca (accessed on 10 October 2021)). Pareto was used as the scaling factor, to make variables comparable to each other. Analysis of Variance (ANOVA) with False Discovery Rate multiple testing correction (FDR) was applied to detect features significantly different after treatment, setting a *p*-value threshold of 0.05.

For each lipid class, we calculated the Unsaturation Index (UI), an estimate of the average number of unsaturations, using the formula: UIy = [Σ (% area lipidx × number of double bonds lipidx)]/100, where lipidx represents each single molecular species belonging to the y lipid class, and the Average Chain Length (ACL), an estimate of the average length of acyl chains, using the formula: ACLy = [Σ (% area lipidx × total number of acyl chains—carbon atoms of lipidx)]/100.

### 2.6. Statistical Analysis

Data are presented as number (%) for dichotomous variables, mean ± standard deviation for continuous variables with a normal distribution and median (interquartile range [IQR]) for non-parametric continuous variables.

## 3. Results

Out of the 133 patients screened, 108 were excluded because of at least one exclusion criterion. The other 25 subjects (13 males and 12 females, 100% Caucasian, mean age 51.5 ± 14.5 years, mean body mass index (BMI), 26 ± 4.8 kg/m^2^) with a diagnosis of FH (21 heterozygotes for the LDL receptor mutation, 1 double heterozygote for 2 different mutations of the LDL receptor and 3 subjects wild-type genetics) met the criteria and were enrolled in the study. Their total cholesterol was 279.6 ± 69.9 mg/dL; triglycerides, 107.5 mg/dL (IQR: 79.3–146.3); HDL-C 53 ± 12.93 mg/dL; LDL-C 201 ± 69.50 mg/d; Lap(a) 69.3 (IQR:19.8–83.4) mg/dL, and LDL score, 6.6 (IQR:4.65–11.7). None of them had received a treatment with PCSK-9 inhibitor before study entry. Their alanine aminotransferase (ALT), aspartate aminotransferase (AST) and creatin-phosphokinase values were 24 (IQR:21–26), 27 (IQR: 21.3–38.8) and 110 (IQR: 90–171) IU/L, respectively. Data on gender, age, previous and current medical conditions, current and past lipid-lowering therapy and vascular risk factors (VRFs) were collected for each patient. Hypertension was present in 12/25 (48%) of them, obesity in 13/25 (52%), diabetes mellitus and smoking habit in 2/25 (8%), respectively. Twenty patients (80%) were under statin treatment; whereas five reported statin intolerance. Among the 20 statin-treated patients, 1 was on simvastatin 40 mg, 3 on rosuvastatin 40 mg, 5 on rosuvastatin 20 mg, 1 on pravastatin 40 mg, 1 on fluvastatin 40 mg, 5 on atorvastatin 40 mg, 3 on atorvastatin 80 mg and 1 on monakolin k. Ezetimibe was used as a co-treatment in 10 subjects (40%) and as a stand-alone treatment in the 5 subjects (20%) with statin intolerance. Antiplatelet drugs were in use in 9 subjects (36%). A history of vascular events was present in 7 subjects (28%, coronary artery disease in 6 cases, ischemic stroke in 1 case). Height was measured to the nearest 0.1 cm. After emptying the bladder, body weight was assessed by an electronic beam scale with digital readout to the nearest 0.1 kg, and with the subjects standing barefoot and wearing light indoor clothing. Diagnosis of familial FH was based on the Dutch Lipid Clinic Network Score (mean Dutch lipid score was 9.3 ± 3.6), and genetics identified causative mutations, including those of LDL receptor, apo-B and PCSK-9 genes [18].

### 3.1. Serum Cholesterol Profile and Lipoprotein Levels after 4- and 12-Week Treatment

As expected, after 4-week treatment with PCSK-9 inhibitor (T4w), all patients showed an improved lipid profile. TC was reduced from 279.6 ± 69.9 mg/dL to 177.3 ± 68.2 mg/dL (*p* < 0.001), LDL from 201.0 ± 69.5 mg/dL to 100.2 ± 70.5 mg/dL (*p* < 0.001), and Lp(a) from 69.3 mg/dL (IQR: 19.8–83.4) to 52.3 mg/dL (IQR: 17.0–80.6) (*p* = 0.002). The % of patients with high-Lp(a) was 56.5% both at baseline and at T4w. LDL-C target was achieved in 14/25 patients (56%), being more frequently found in patients receiving combined treatment statin+PCSK-9 inhibitor (73.7%). LDL diameter changed from 268.3 ± 2.99 Å to 270.4 ± 3.02 Å (*p* = 0.003), an event associated with LDL score reduction from 6.60 (IQR: 4.65–11.7) to 3.0 (IQR: 0.8–7.9) (*p* = 0.037). Correlation between changes in LDL diameter and LDL score was rho = −0.770, *p* < 0.001. TGL (from 107.5 (IQR: 79.3–146.3) mg/dL to 97.0 (IQR: 62.5–119.5) mg/dL (*p* = 0.157)) and HDL (52.5 ± 12.9 mg/dL at T0 vs. 53.2 ± 12.8 mg/dL at T4w (*p* = 0.616)) were not affected by 4-week treatment. 

After 12 weeks of treatment with the PCSK-9 inhibitor (T12w), patients reported approximately 34.9% lower levels of TC (from 279.6 ± 69.9 mg/dL to 181.8 ± 64.8 mg/dL, *p* < 0.001) and approximately 48.7% (201.0 ± 69.5 mg/dL to 103.0 ± 58.0 mg/dL, *p* < 0.001) reduction in LDL, with a slight increase in HDL (from 52.5 ± 12.9 mg/dL to 55.0 ± 12.6 mg/dL (*p* = 0.065)). LDL-C target was achieved in 15/25 patients (60%). LDL diameter changed from 268.3 ± 2.99 Å to 270.5 ± 2.40 Å (*p* = 0.004) with LDL score changing from 6.60 (IQR: 4.65–11.7) to 3.7 (IQR: 0.37–4.9) (*p* = 0.001). The correlation between changes in LDL diameter and LDL score was rho = −0.730, *p* < 0.001. TGL did not significantly change (from 107.5 mg/dL, IQR: 79.3–146.3 mg/dL to 109.0 mg/dL, IQR: 73.5–146.0 mg/dL, *p* = 0.607). In contrast, Lp(a) changed from 69.3 mg/dL (IQR: 19.8–83.4) to 41.7 mg/dL (IQR: 13.5–80.6) (*p* = 0.001). However, the % of patients with high-Lp(a) was only slightly reduced (from 56.5% to 45.5%, *p* = 0.462). Patients enrolled in the study reported no adverse events during the entire follow-up. 

### 3.2. Effects of pcsk9 Inhibitors on Plasma Lipidome

After the 12-week treatment, 26 lipid features belonging to phosphatidylcholine (PC), sphingomyelin (SM), ceramide (CER), cholesteryl ester (CE), triacylglycerol (TG) and phosphatidylinositol (PI) classes, were significantly reduced from baseline, while 4 species (3 CER and 1 PI) were increased after the treatment (Table 1).

Despite individual differences, lipid reduction was graded between baseline, T4w and T12w (data not shown). In particular, five polyunsaturated diacyl PC (PC 34:2, PC 36:2, PC 36:4, PC 37:6 and PC 38:4), two ether/vinyl ether forms (PC O-32:0 and (O-36:3) (P-36:2)) and one oxidized molecule (PC 34:1 (OH)) were reduced by the treatment. The analysis also revealed that these five reduced diacyl-PCs are a large proportion of total PC at baseline (range:38.6 to 49.2%; data not shown). Very interestingly, variance with its oxidized form PC 34:1 (OH), PC 34:1 (10.4 to 20.5% of total PC) was not significantly affected by PCSK9 inhibitor treatment. Seven SM, 5 CER and HEX-CER were significantly reduced at T12w. Contrary to what described for PC, SM and CER, reduction seems to be a general trend given that both medium- and long-chain saturated and unsaturated species were affected. The only exception was the two long-chain monounsaturated CER (d43:1 and d44:1) that were increased together with the S-HEX-CER (d40:2). Two PI species showed opposite trends as compared to baseline data: PI 38:5 was reduced while the PI 40:5 was increased. Consistent with the described TC reduction, CE 18:2 and CE 20:4, together accounting for 87.6% to 98.2% of total CE, were strongly reduced by the treatment. In contrast, neutral lipids DAG and TG were unaffected, indicating that the treatment with PCSK-9 inhibitor does not affect TG both quantitatively and qualitatively. Two exceptions are represented by odd-chain TG that were reduced. However, this is only a minute proportion of total TG (0.7 to 3.0%, data not shown). Although PE were not significantly affected, it displayed shorter and more saturated fatty acyl chains compared to baseline (qualitative changes, Table 2). Likewise, compared to baseline, the unsaturation index of HEX-CER and lactosyl-(LAC)-CER was reduced.

## 4. Discussion

PCSK-9 inhibitor treatment has been the target of several studies showing significant reductions in TC, LDL and TG [19,20,21,22,23]. Treatment with Evolocumab 140 mg or Alirocumab 150 mg led to overall reductions of LDL-C from 41% to 70% compared [19,20,21,22,23]. In line with these results, we reported significant reduction in TC and LDL, but not for TG.

TG were slightly diminished after 4 weeks’ treatment and returned to baseline levels at T12. Although it is well established that PCSK-9 inhibitors significantly lower LDL-C concentration, some data seem to suggest modest improvement in the concentrations of TG and HDL-C as shown in results of RCTs [24]. However, no dedicated studies were performed on this specific outcome. In light of this, we cannot surely attribute the slight change in TGs observed after 4 weeks of treatment to PCSK9 inhibition.

So far, only two studies (one in humans [9], the other in humans and mice [25]) reported the effects of treatment with PCSK-9 inhibitor at the level of individual lipid species. In accordance with Hilvo et al [9], our lipidomics analysis revealed a significant reduction in SM and CER. However, we also found reductions in PC, CE, LAC-CER, HEX-CER and of some PI after 12 weeks of treatment with Evolocumab 140 mg. Among such lipid classes, SM, CER and CE were maximally affected by the treatment. Very interestingly, two long-chain monosaturated CER (d43:1 and d44:1), one S-HEX-CER (d40:2) and one PI (PI 40:5) were increased after 12-week treatment. Supporting the results of the present study, a clear reduction in SM, LAC-CER and CE in plasma has been demonstrated in knockout (KO) mice for PCSK9 and in human carriers of the loss-of-function (LOF) mutation of PCSK9 [10]. 

Raised levels of SM and CER are independently associated with cardiovascular risk [10,26,27]. LAC-CER is thought to play a role both in intimal medium proliferation and in coronary endothelial dysfunction through the reduction in nitric oxide production [28]. Moreover, plasma CER levels predict cardiovascular death in patients with stable coronary artery disease and acute coronary syndromes beyond LDL-cholesterol [26]. Studies in mice show that SM are involved in the early phases of atherosclerosis through mechanisms involving signal transduction pathways that affect the proliferation of endothelial cells and the formation of foam cells [27,29]. Studies on KO mice for LDL-R, in which the bone marrow of KO mice for the enzyme sphingomielin synthase 1 (SMS) was transplanted, showed a reduction in atherosclerosis at the level of the aorta. The absence of the SMS enzyme in the monocyte macrophage system reduces atherosclerosis progression, arguing for a role of SM in atherosclerosis independent of the LDLR [30].

In keeping with this, activation of sphingomyelinase by the activated endothelium and by plaque macrophages is implicated in the retention and aggregation of apoB-lipoproteins and, hence, in facilitating adherence of apoB-lipoproteins to the arterial matrix, a key mechanism in atherogenesis [31]. Moreover, CER plays a central role in sphingolipid metabolism. Once synthesized, CER triggers the activity of sphingomyelinase, thus releasing oleic acid by cytosolic phospholipase A 2 (cPLA 2), and in turn increasing CE production [32].

The activation of cPLA 2 in platelets is implicated in the release of arachidonic acid, indicating its potential role in platelet aggregation [33,34,35]. 

Together, the pathophysiological mechanism through which PCSK-9 inhibition and the consequent up-regulation of LDL-R modify the lipidome seems to be related to an indirect mechanism. Oxidized LDL acts as a stimulus for the de novo synthesis of SM and CER through the activation of the key enzyme sphingomyelin synthase [27]. Experimental evidence shows that the de novo synthesis of ceramides is involved in cholesterol esterification [27]. Together with oxidative modifications in LDL particles, cholesterol esterification exerts antigenic effects and, by activating both innate and adaptive immunity [36], promotes atherosclerosis-associated inflammation. The present data suggest that oxLDL stimulate the de novo synthesis of ceramide, which promotes the hydrolytic activity of cPLA 2, to release oleic acid, thus increasing cholesterol ester production [27]. 

A limitation of the present report is the lack of determination of antibodies against oxidized LDL (oLAb) that play an important role in the modulation of acute coronary syndrome. However, due to the untargeted approach employed, the present report was hypothesis-generating and needs to be confirmed with specifically designed experiments with targeted analysis. Nevertheless, the present data provide the rationale for such studies. 

## 5. Conclusions

Overall, LDL reduction appears to be an important factor in the modification of phospholipids. The improved VLDL catabolism and the related reduction in apo-B containing lipoproteins is likely to be involved in lipidomic changes in patients treated with PCSK-9i [10]. These data rule out direct mechanisms of PCSK-9 inhibition on lipidomic modifications and argue for a multifaceted system leading to vascular improvement in users of PCSK-9 inhibitors, in line with clinical data showing subclinical atherosclerosis regression documented in patients receiving a PCSK-9 inhibitor [37,38]. However, considering that an untargeted lipidomic approach is a hypothesis-generating technique, a targeted approach based on lipid fraction isolation would provide further insights on this topic.

## Figures and Tables

**Table 1:** Lipid molecular species significantly differing from baseline values according to ANOVA analysis.

Lipid Molecular Species	Lipid Class	Adjusted *p*-Value	Change
PC 34:2 [M+H]+	PC	0.0042255	↓
PC 36:2 [M+H]+	PC	0.0067707	↓
PC 36:4 [M+H]+	PC	0.020646	↓
PC 37:6 [M+H]+	PC	0.0067707	↓
PC 38:4 [M+H]+	PC	0.04987	↓
PC (O-32:0) [M+H]+	PC	0.043201	↓
PC (O-36:3) (P-36:2) [M+H]+	PC	0.011944	↓
PC 34:1 (OH) [M+H]+	PC	0.039059	↓
SM (d33:1) [M+H]+	SM	0.043486	↓
SM (d34:0) [M+H]+	SM	0.00057655	↓
SM (d38:2) [M+H]+	SM	0.011944	↓
SM (d40:2) [M+H]+	SM	0.011944	↓
SM (d42:1) [M+H]+	SM	0.0071241	↓
SM (d42:2) [M+H]+	SM	0.00057655	↓
SM (d42:3) [M+H]+	SM	0.011944	↓
TG 47:3 [M+Na]+	TG	0.017528	↓
TG 51:1 [M+Na]+	TG	0.016612	↓
CE 20:4 [M+H]+	CE	0.041264	↓
CE 18:2 [M+H]+	CE	0.041264	↓
CER (d33:1) [M+HCOONH4]−	CER	0.0071241	↓
CER (d38:1) [M+HCOONH4]−	CER	0.043201	↓
CER (d40:0) [M+HCOONH4]−	CER	0.012228	↓
CER (d42:0) [M+HCOONH4]−	CER	0.016612	↓
CER (d44:0) [M+HCOONH4]−	CER	0.043486	↓
HEX-CER (d42:2) [M+HCOO]−	CER	0.024282	↓
PI 38:5 [M−H]−	PI	0.020376	↓
CER (d43:1) [M+HCOO]−	CER	0.0065246	↑
CER (d44:1) [M+HCOO]−	CER	0.043288	↑
S-HEX-CER (d40:2) [M−H]−	CER	0.047847	↑
PI 40:5 [M−H]−	PI	0.043486	↑

*p*-values are reported after FDR correction. ↓: lipid species decreased from baseline; ↑: lipid species increased from baseline.

**Table 2:** Average Chain Length (ACL) and Unsaturation Index (UI) for each lipid class at each time point.

	ACL				UI			
Lipid Class	T_0_	T_4_	T_12_	(*p*-Value)	T_0_	T_4_	T_12_	(*p*-Value)
LPC	17.5178	17.4820	17.4140	0.644125	0.2203	0.2147	0.2000	0.683932
PC	35.6470	35.6681	35.6783	0.6839315	0.9930	1.0043	1.0048	0.81635
LPE	19.7342	19.7836	19.8026	0.881867	0.0197	0.0141	0.0127	0.906363
PE	37.4263	37.3464	37.2701	0.024802 *	1.6802	1.5942	1.5630	0.002333 *
PI	37.5724	37.5341	37.5332	0.698246	1.3583	1.3476	1.3451	0.771916
SM	37.9698	38.0673	38.0144	0.286808	1.4340	1.4550	1.4485	0.113967
CER	41.0776	41.0955	40.9025	0.4175	1.2107	1.2265	1.2168	0.609739
HEX-CER	38.8486	38.6879	38.8002	0.103829	1.2876	1.2578	1.2498	0.013582 *
LAC-CER	34	34	34	1	1.0585	1.0696	1.0746	0.043693 *
S-HEX-CER	35.3425	35.7382	35.4966	0.04639 *	1.6535	1.6796	1.6500	0.527907
CE	18.8499	18.8671	18.8674	0.924687	2.7793	2.7927	2.7976	0.921739
DAG	36.3020	36.2523	36.1798	0.343138	2.6973	2.6425	2.5910	0.088299
TG	52.2924	52.2627	52.2426	0.938821	3.3240	3.2965	3.2587	0.795857

*p*-value after ANOVA. *: statistically significant.

## Data Availability

Not applicable.

## Abbreviations

CE	cholesteryl ester
CER	ceramide
HEX-CER	glucosyl/galactosyl-ceramide
LAC-CER	lactosyl-ceramide
S-HEX-CER	3-O-sulfogalactosylceramide
DAG	diacylglycerol
TG	triacylglycerol
PC	phosphatidylcholine
PE	phosphatidylethanolamine
LPC	lysophosphatidylcholine
LPE	lysophospatidylethanolamine
PI	phosphatidylinositol
SM	sphingomyelin
TAG	triacylglycerol
